# GAD65 haplodeficiency conveys resilience in animal models of stress-induced psychopathology

**DOI:** 10.3389/fnbeh.2014.00265

**Published:** 2014-08-07

**Authors:** Iris Müller, Kunihiko Obata, Gal Richter-Levin, Oliver Stork

**Affiliations:** ^1^Department of Genetics and Molecular Neurobiology, Institute of Biology, Otto-von-Guericke UniversityMagdeburg, Germany; ^2^National Institute for Physiological SciencesOkazaki, Aichi, Japan; ^3^Department of Neurobiology and Ethology and Department of Psychology, Institute for the Study of Affective Neuroscience, University of HaifaHaifa, Israel; ^4^Center for Behavioural Brain SciencesMagdeburg, Germany

**Keywords:** glutamate decarboxylase, juvenile trauma, social isolation, stress resilience, mutant mouse, posttraumatic stress disorder

## Abstract

GABAergic mechanisms are critically involved in the control of fear and anxiety, but their role in the development of stress-induced psychopathologies, including post-traumatic stress disorder (PTSD) and mood disorders is not sufficiently understood. We studied these functions in two established mouse models of risk factors for stress-induced psychopathologies employing variable juvenile stress and/or social isolation. A battery of emotional tests in adulthood revealed the induction of contextually generalized fear, anxiety, hyperarousal and depression-like symptoms in these paradigms. These reflect the multitude and complexity of stress effects in human PTSD patients. With factor analysis we were able to identify parameters that reflect these different behavioral domains in stressed animals and thus provide a basis for an integrated scoring of affectedness more closely resembling the clinical situation than isolated parameters. To test the applicability of these models to genetic approaches we further tested the role of GABA using heterozygous mice with targeted mutation of the GABA synthesizing enzyme GAD65 [GAD65(+/−) mice], which show a delayed postnatal increase in tissue GABA content in limbic and cortical brain areas. Unexpectedly, GAD65(+/−) mice did not show changes in exploratory activity regardless of the stressor type and were after the variable juvenile stress procedure protected from the development of contextual generalization in an auditory fear conditioning experiment. Our data demonstrate the complex nature of behavioral alterations in rodent models of stress-related psychopathologies and suggest that GAD65 haplodeficiency, likely through its effect on the postnatal maturation of GABAergic transmission, conveys resilience to some of these stress-induced effects.

## Introduction

Exposure to severe or prolonged stress can evoke multifaceted psychopathologies such as post-traumatic stress disorder (PTSD) and mood disorder. PTSD emerges in the aftermath of a potentially life threatening event and is characterized by anxiety, hyperarousal, generalized fear and intrusive memories, as well as depressive symptoms (reviewed in Siegmund and Wotjak, 2006; Yehuda and LeDoux, 2007). However, of those who experience a given traumatic situation only about 20–30% subsequently develop the disorder (Zohar et al., 2008). Both genetic disposition and environmental conditions such as poor social support and/or childhood maltreatment comprise important risk factors (Heim and Nemeroff, 2001). In recent years, extensive research with Pavlovian fear conditioning paradigms in rodents has provided insight into cellular and molecular processes of fear learning that may also account for processes in disease development (Johansen et al., 2011). In addition, animal models have been developed that recapitulate key features of stress-induced psychopathologies and emphasize both severe stress experiences and the role of social neglect (Avital et al., 2006; Tsoory et al., 2007; Pibiri et al., 2008; Bazak et al., 2009). Finally, the need for an integrative behavioral analysis of the complex stress-evoked phenotype in such animal models has been recognized (Horovitz et al., 2012).

Evidence suggests that GABAergic neurotransmission plays an important role in traumatic fear-induced pathology. For instance, a polymorphism in the GABA_A_-receptor β 3 subunit gene has been associated with increased symptom severity, among which range anxiety, social dysfunction and depression in a population of PTSD-patients (Feusner et al., 2001). PTSD-patients also had significantly lower GABA-plasma levels immediately after as well as 1-year after trauma exposure compared to trauma-exposed healthy subjects (Vaiva et al., 2004, 2006). Some (Drake et al., 2003), but not all (Davidson et al., 2007; Davis et al., 2008) GABA-agonistic drugs represent an effective therapy of PTSD. Similarly, GABA-related drugs have also been implicated in the treatment of mood disorders (Leung and Xue, 2003; Kendell et al., 2005). In rats, stress experience reduces GABA-stimulated chloride uptake in amygdala and frontal cortex (Martijena et al., 2002) and juvenile stress alters the GABA_A_-receptor subunit composition in the amygdala (Jacobson-Pick et al., 2008). Moreover, benzodiazepine infusion into the rat basolateral amygdala prevents stress-induced facilitation of fear conditioning (Rodríguez Manzanares et al., 2005).

We have previously observed hyperarousal and anxiety, as well as fear generalization and resistance to extinction in mice deficient for the GABA synthesizing enzyme glutamic acid decarboxylase (GAD)65 (Stork et al., 2000a; Bergado-Acosta et al., 2008; Sangha et al., 2009), suggesting that GABA synthesis is a critical factor for several PTSD-related phenomena. GAD65(−/−) mice show a reduction of GABA tissue content in the adult and a delayed replenishment of GABAergic vesicles, which results in enhanced hippocampal long-term potentiation (Kash et al., 1999; Stork et al., 2000a). However, baseline inhibition appears to be unaffected, due to compensatory changes, such as increase of vesicular GABA transporter and GABA uptake into synaptic vesicles (Wu et al., 2007). Interestingly, the observed deficit in GABA tissue content of GAD65(−/−) mice develops during the 2nd postnatal month, suggesting a function of GAD65 in brain maturation during adolescence. In heterozygous GAD65 mutants, the maturation of the GABAergic system is delayed, as tissue concentrations of transmitter reach wild type levels only after the 3rd postnatal month (Stork et al., 2000a).

Given the importance of juvenile development for PTSD, we hypothesized that this GAD65-mediated postnatal maturation of the GABA system may be of particular relevance for the disease. We, therefore, investigated the behavior of wild type and GAD65 haplodeficient mice in two established models of stress-induced psychopathologies, triggered by variable juvenile stress and/or protracted social isolation. Both models, although differing in stressor type, intensity and duration, have previously been shown to involve changes in GABAergic functions (Jacobson-Pick et al., 2008; Pibiri et al., 2008) and may thus be suitable to detect the effect of genetic modifications in this system. To determine the mutant's response to stress without narrowing down to only one symptom we examined the behavioral outcome with a set of behavioral tests covering relevant behavioral domains. A factor analysis was applied to systematically evaluate the outcome of the stress exposure. This allowed us to discriminate anxiety, arousal and depression-related changes in both PTSD models and to identify an unexpected resilience to traumatic stress conveyed by the GAD65 haplodeficiency.

## Materials and methods

### Animals

Male heterozygous GAD65(+/−) mice, bred on a C57BL/6N (Tac) background (M and B Taconic), and their wild type littermates were used. Experimental animals were obtained from GAD65(+/+) X GAD65(+/−) breeding and genotyped by allele-specific polymerase chain reaction as described previously (Stork et al., 2000a). Mice were kept in our animal facility in groups of two to six under an inverted light/dark cycle with lights on at 7:15 pm and off at 7:15 am. They had *ad libitum* access to food and water. All experiments were approved by the Landesverwaltungsamt Sachsen-Anhalt (TV Nr 203.h-42502-2-887 OvGMD and 203.h-42502-2-939 UniMD).

Mice were randomly divided into one of four groups:

*Variable stress (VS)*: Mice received a variable stress protocol from postnatal day 24–26 adopted from Tsoory et al. (2008). On P24 mice were immobilized for 30 min using a 20 ml plastic tube (length: 9 cm, diameter: 2 cm; Braun Melsungen, Germany), with holes at the front allowing animals to breathe freely. On the following day mice were exposed three times for 30 min, at 1 h intervals, to a bright light (400 lux) on a circular, 105 cm elevated platform (diameter: 14.5 cm; Greiner, Frickenhausen, Germany). And on P26 mice had to swim for 15 min in 24 ± 2°C warm water in a bucket of 16 cm diameter. Restraint and forced swimming took place under red light conditions (<5 lux). Animals were returned to their home cage and group housed until P107, when they were separated for 5 days before behavioral testing.

*Isolation stress (IS)*: Mice were isolated at P24 and kept single in standard home cages until the end of behavioral tests.

*Combined variable and isolation stress (CS)*: Mice were exposed to the variable stressors and were socially isolated as described above.

*Control*: Mice were left undisturbed in littermate groups of 3–5 until P107, when they were separated in preparation of the behavioral testing.

### Behavioral tests

Behavioral testing commenced on P112 and was carried out by an experimenter who was blind to the pretreatment and the genotype of the animals. All animals underwent every test in the order listed below. Order of the tests was chosen according to stress level of the tests and behavioral relevance for PTSD-like features, thus employing anxiety tests before and after fear conditioning.

#### Open field

In the open field (OF) test mice were placed in the center of a square arena (50 × 50 cm) and allowed to explore the new environment for 20 min in red light. Time in the center (25 × 25 cm) was recorded to assess anxiety and the distance the mice moved was tracked as a parameter of activity (ANY-maze™ Video Tracking System, version 4.50, Stoelting Co., Wood Dale, USA).

#### Elevated plus maze

Mice were tested on the elevated plus maze (EPM) for 5 min under low light conditions (10 lux). Entries to open and closed arms were recorded as measures of anxiety and activity using the ANY-maze™ system (Rehberg et al., 2010; Albrecht et al., 2013).

#### Fear conditioning

The fear conditioning apparatus (TSE, Bad Homburg, Germany) and the protocol used are described in Laxmi et al. (2003). In brief, the mice were confronted with four adaptation sessions on 2 days each containing four tone presentations (CS−: 2.5 kHz, 10 s, ISI 20 s) followed by a training session on the next day with 3 tone shock pairings (CS+: 9 s, 10 kHz, 0.4 mA, 1 s; ISI 20 s). Two weeks later a retrieval in the neutral context (4 × CS−, 4 × CS+, 10 s, ISI 20 s) and one day later in the shock context (context) were performed. In all of these, stimulus presentations were preceded by a 2 min stimulus-free interval at the beginning and followed by the same at the end of the session Thus 2 min intervals of CS−, CS+ and context were analyzed for freezing (complete immobility except for respiratory movements) and automatically recorded online as indicator for learning by a photo beam system.

#### Light/dark test

Animals were placed in the light compartment (100 lux) of a standard light/dark test and their behavior was recorded for 5 min using a photo beam system (TSE). The activity (movements at a velocity of more than 3 cm/s) in the light and dark compartments was recorded as measures of anxiety (% activity in the light) and activity (cumulative activity in light and dark; (Stork et al., 2000a; Laxmi et al., 2003).

#### Social interaction

Social interaction was tested in a standard 3-compartment chamber (40 × 20 cm) with one circular containment tube (8 cm in diameter and with holes spaced 1 cm apart) in the outer compartments. In 5 min adaptation mice were allowed to explore the empty chamber. Then a young male mouse was put in either cylinder and interactions with the tubes were manually scored (Albrecht and Stork, 2012).

#### Tail suspension

Mice were suspended on a cylinder for 5 min with a tape wrapped around the tail. Time immobile was recorded manually as a measure for depression-like behavior (Cryan et al., 2005).

### Statistical analysis

Two-Way ANOVAs (genotype and stress group) were performed followed by Fisher's LSD tests for *post-hoc* comparisons, if significant group effects or significant interaction effects were obtained. In cases of only significant group effects, LSD-tests were performed only within one genotype. In cases of only significant genotype effects, *t*-tests were carried out. To avoid false positive effects and to ensure data homogeneity, outliers were identified using the Dean and Dixon test and excluded from analysis for the respective test. In addition we performed a factor analysis with Varimax rotation (Table 1, Table S1) on all parameters evaluated in the test battery. Factor extraction was validated with a Quartimax rotation (Table S2). To test genotype and stress group effects on whole factors, parameters were z-transformed to allow merging of measures with different units. Parameters that loaded high (above 0.5) on the same factor were averaged to form the indices, if necessary they were multiplied by (−1) before averaging for achieving the same polarity.

**Table 1:** **Factor loadings of the analyzed parameters for every factor extracted by factor analysis**.

**VARIMAX-rotation**	**Rotated component matrix**				
	**1**	**2**	**3**	**4**	**5**
OF: distance (m)	0.093	**0.867**	−0.114	−0.078	−0.053
OF: center time (s)	−0.129	0.011	**0.807**	−0.083	0.004
EPM: total arm entries	0.059	**0.833**	0.026	−0.061	0.083
EPM: % open arm entries	0.39	−0.058	**0.656**	0.212	−0.117
FC: shock context_freezing (s)	−**0.506**	−0.242	0.424	0.266	0.07
FC: CS− freezing (s)	0.028	0.089	−0.127	0.08	**0.874**
FC: CS+ freezing (s)	−0.42	−0.23	0.324	−0.072	**0.515**
LD: % activity in light	**0.865**	−0.124	−0.039	−0.202	0.097
LD: total activity (s)	**0.735**	0.327	0.164	0.102	−0.111
SI: % time of mouse contacts	−0.034	0.052	−0.038	−**0.817**	−0.285
TS: time immobile (s)	−0.236	−0.112	0.027	**0.679**	−0.365

*The first factor is represented by context freezing and post-conditioning activity- and anxiety measures. Pre-conditioning activity and anxiety were shown to be independent from each other (factors 2 and 3). Social interaction and depression measures loaded on the same factor (factor 4). Cue-related fear memory displayed high correlations to the fifth factor. Factor loadings above 0.5 are considered high loadings and highlighted in bold. Extraction Method: Principal Component Analysis. Rotation Method: Varimax with Kaiser Normalization, rotation converged in 8 iterations*.

## Results

### Open field

**Figure 1:**
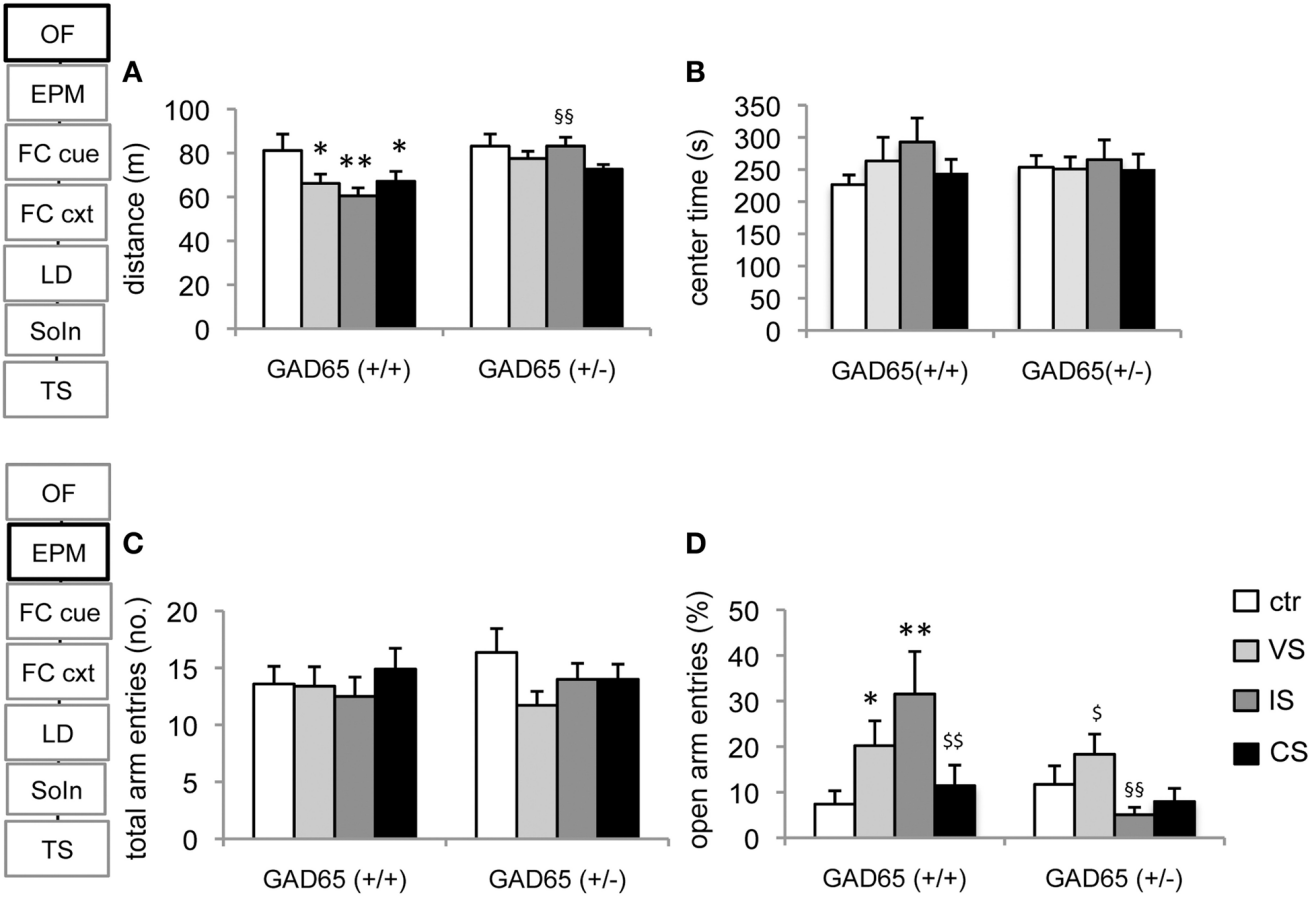
**Open field and elevated plus maze. (A)** All three stress procedures led to a significant reduction in activity in the open field in GAD65(+/+) but not in GAD65(+/−) mice in the OF. Socially isolated wild type mice differed significantly from their heterozygous littermates of the same group. **(B)** No behavioral alterations were observed with respect to the time spent in the center. **(C)** In the EPM no differences were observed in total arm entries in either genotype or stress group. **(D)** Heterogeneous results were obtained in the percentage of open arm entries. In GAD65(+/+) mice VS and IS increased open arm entries, but mice confronted with a combination of both stressors were indistinguishable from controls. In GAD65(+/−) mice social isolation reduced open arm exploration that reached significance when VS and IS mice were compared. Data are mean ± s.e.m. ^*^p < 0.05, ^**^p < 0.01 compared to ctr of the same genotype, ^§§^*p* < 0.01 compared to GAD65 (+/+) of the same stress group, ^$^*p* < 0.05, ^$$^*p* < 0.01 compared to IS of the same genotype.

For the distance traveled in the open field, a significant effect of genotype [TWA, *F*_(1, 80)_ = 8.463, *p* = 0.005] and stress group became apparent [*F*_(3, 80)_ = 2.85, *p* = 0.043]. In wild type mice each of the stressors induced a significant reduction in activity [variable stress (VS): *p* = 0.038, isolation stress (IS): *p* = 0.006, combined stress (CS): *p* = 0.045] compared to controls. These changes generally failed to reach significance in GAD65(+/−) mice and socially isolated GAD65(+/−) differed significantly from the corresponding group of GAD65(+/+) mice (*p* = 0.006). On the other hand, no significant effect on center exploration of the open field could be observed (Figures 1A,B).

### Elevated plus maze

Concerning open arm exploration, significant genotype [*F*_(1, 75)_ = 4.562, *p* = 0.03] and stress group effects [*F*_(3, 75)_ = 2.815, *p* = 0.045] as well as genotype x stress group interaction [*F*_(3, 75)_ = 3.813, *p* = 0.013] were observed. In GAD65(+/+) mice, enhancement of open arm exploration was observed in the VS-group (*p* = 0.046) and the IS-group (*p* = 0.001), compared to control. Strikingly, the increase was abolished in the combined CS-group (*p* = 0.006, compared to IS). In GAD65(+/−) mice, the IS group failed to increase open arm exploration (*p* = 0.046, compared to VS) and differed from the behavior of the corresponding GAD65(+/+) mice (*p* = 0.001). No significant effects of genotype or treatment were evident with respect to total arm entries (Figures 1C,D).

### Fear conditioning

Our data reveal a significant stimulus (CS−, CS+, context) effect on memory retrieval [*F*_(1.775, 131.319)_ = 86.8, *p* < 0.001], a significant main effect for stressor type [*F*_(3, 74)_ = 4,698, *p* = 0.005] and a significant interaction between memory type and stress group [*F*_(5.324, 131.319)_ = 3.786, *p* = 0.003].

#### Within-group comparisons

In GAD65(+/+) mice, freezing to the CS+ was significantly higher than freezing to the CS− in all experimental groups (control: *p* = 0.001; VS: *p* = 0.03; IS and VS+IS: *p* < 0.001). Freezing to the context was only elevated of CS− levels in the stress groups (VS: *p* = 0.032; IS: *p* = 0.001 and CS: *p* < 0.001), but not in the control group. Only the latter group displayed significantly increased freezing levels, when CS+ was compared to the context (*p* = 0.001). In GAD65(+/−) mice, freezing to the CS+ was significantly higher than freezing to the CS− in all experimental groups (control: *p* = 0.001; VS, IS and CS: *p* < 0.001). Freezing to the context compared to the CS− was only elevated in groups IS (*p* = 0.005) and CS (*p* = 0.001), but neither in control nor in the VS group. Freezing levels to the context compared to the CS+ reached significance in the VS (*p* = 0.01) and the VS+IS (*p* = 0.016) groups.

#### Between group comparisons

In GAD65(+/+) mice all three stressors induced a contextual generalization compared to controls (control vs. VS: *p* = 0.033, control vs. IS: *p* = 0.004, control vs. CS: *p* = 0.002). In GAD65(+/−) however a significant difference was obtained only between the VS− and the IS− group (*p* = 0.043).

**Figure 2:**
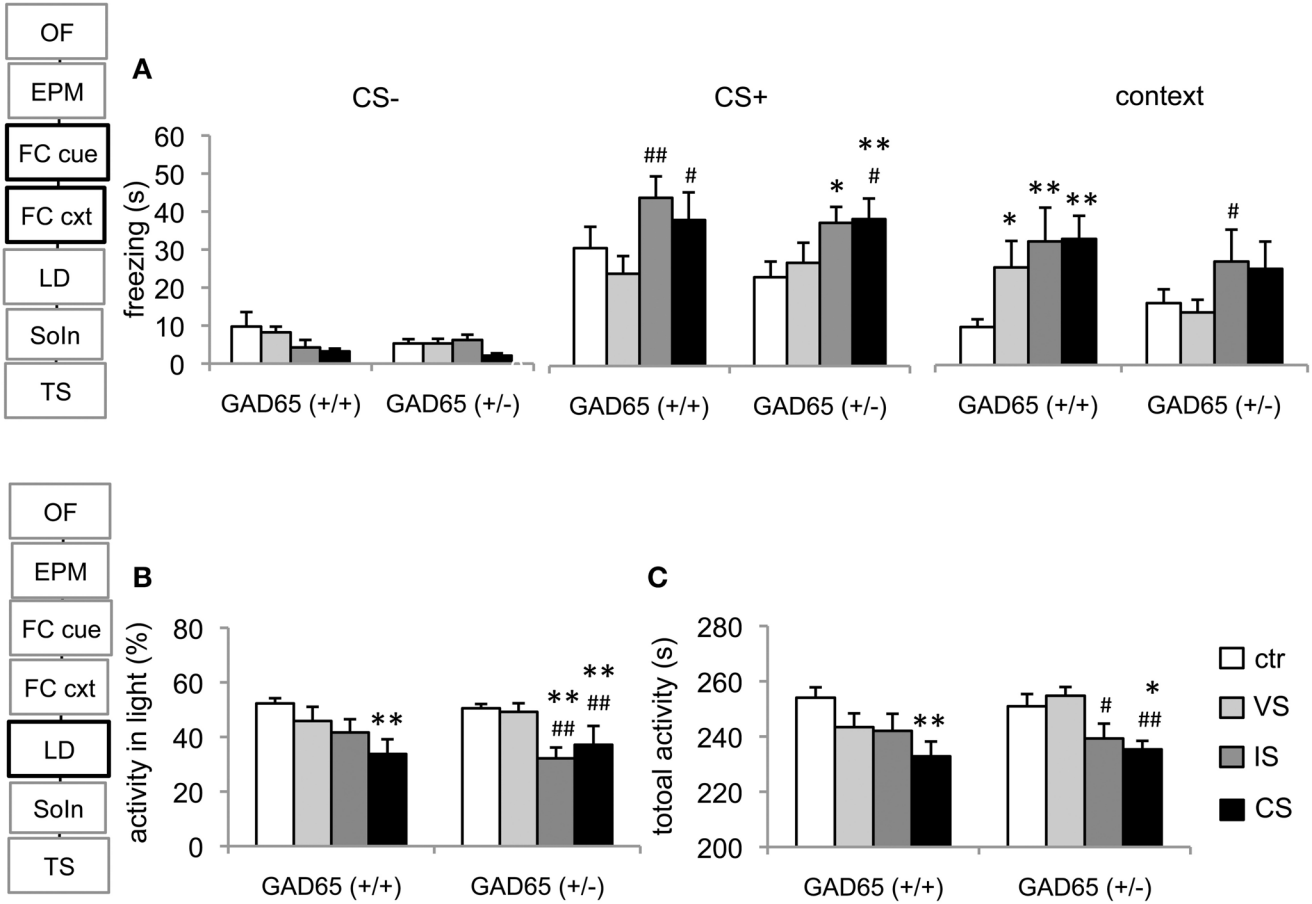
**Conditioned fear and light-dark avoidance test. (A)** Cued fear memory did not generalize to a neutral tone (CS−) in response to stress experience or genotype. Social isolation increased freezing to the conditioned tone (CS+) in both genotypes, reaching statistical significance when wild type IS and CS mice were compared to VS mice. IS and CS heterozygous mice differed significantly from the ctr-group and the CS-group also differed from VS mice. In GAD65(+/+) mice all three stress protocols lead to contextual fear generalization. In contrast, only the IS-group differed from the VS-group in GAD65(+/−) mice. **(B)** In GAD65 wild type mice the three stress protocols lead to a gradual decrease in their activity in the light in the light-dark avoidance test, reaching significance in the CS-group. In GAD65(+/−) mice stressors that contained protracted social isolation significantly decreased activity in the light compartment compared to ctr and VS mice. The latter are indistinguishable from unstressed controls. **(C)** A similar pattern arose in respect to total activity, with wild type mice showing a gradual reduction with stress severity and heterozygous mice displaying a strong dependency on the stressor type. Data are mean ± s.e.m. ^*^p < 0.05, ^**^p < 0.01 compared to ctr of same genotype, ^#^*p* < 0.05, ^##^*p* < 0.01 compared to VS of the same genotype.

The response to the CS+ and CS−, in contrast was largely similar between genotypes: In GAD65(+/+) mice, the IS-group and the CS-group showed increased freezing levels (*p* = 0.009 and *p* = 0.049, respectively) compared to VS. In GAD65(+/−) mice a similar increase was observed in these groups (IS vs. control: *p* = 0.021; CS vs. control: *p* = 0.009; CS vs. VS: *p* = 0.049). No significant difference was seen between groups concerning the response to the CS− (Figure 2A).

### Light/dark test

A significant effect on the activity in the light compartment was observed for stress group [*F*_(3, 78)_ = 13.44, *p* < 0.001], but no effect of genotype [*F*_(1, 78)_ = 0.303, *p* = 0.584] or genotype × group interaction was observed [*F*_(3, 78)_ = 1.729, *p* = 0.168]. *Post-hoc* comparisons in GAD65(+/+) mice revealed significantly less activity in the light of the CS-group than of the control-group (*p* = 0.003). GAD65(+/−) mice displayed reduced activity in the light in the IS and the CS group (*p* < 0.001, compared to both control and VS groups). Moreover, an effect of stressor type was observed on the total activity in this test system [*F*_(3, 80)_ = 7.013, *p* < 0.001]. A gradient was observed in GAD65(+/+) mice across stress groups, with a significant reduction of total activity in the CS-group (*p* = 0.003). GAD65(+/−) mice showed a reduced activity in the IS-group (*p* = 0.056 to control, *p* = 0.013 to VS) and in the CS-group (*p* = 0.01; *p* = 0.002) (Figures 2B,C).

### Social interaction

**Figure 3:**
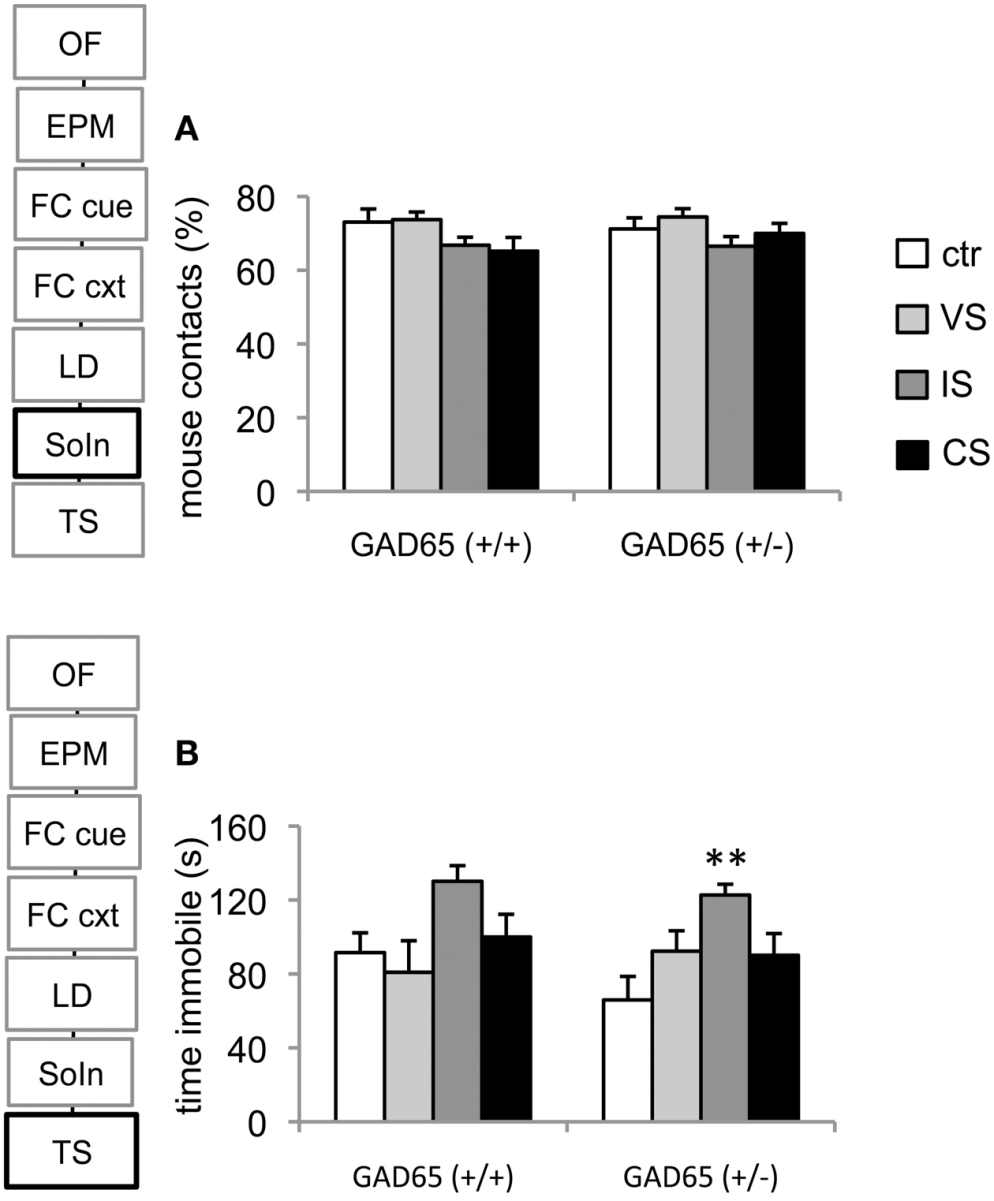
**Social interaction and tail suspension test. (A)** Neither stress nor genotype had an effect on social interaction. **(B)** In both genotypes social isolation produced the strongest effects on depression related behavior, reaching significance in GAD65(+/−) mice. Data are mean ± s.e.m. ^**^p < 0.05 compared to ctr.

Two-Way analysis of variance revealed a significant effect for the stress group [*F*_(3, 72)_ = 3.159, *p* = 0.03], but no effect of genotype [*F*_(1, 72)_ = 0.175, *p* = 0.677] or genotype x stressor interaction [*F*_(3, 72)_ = 0.51, *p* = 0.676]. Although sociability was tendentially reduced in IS exposed groups of both genotypes, no difference could be observed in *post-hoc* comparison (Figure 3A).

### Tail suspension test

A significant stress group effect was also observed concerning immobility in the tail suspension test [Two-Way ANOVA, *F*_(3, 73)_ = 4.43, *p* = 0.006]. Pairwise comparison revealed a preferential effect on the social isolation group (*p* = 0.003 vs. control) in GAD65(+/−) mice. A similar trend was seen in GAD65(+/+) mice but failed to reach significance level (Figure 3B).

### Factor analysis

Factor analysis with Varimax rotation extracted five independent factors that together accounted for 70 % of total behavioral variance (Table S1). Factor separation was validated by repeating the factor analysis with Quartimax rotation (Table S2).

#### Factor 1: generalized contextual fear and anxiety

**Figure 4:**
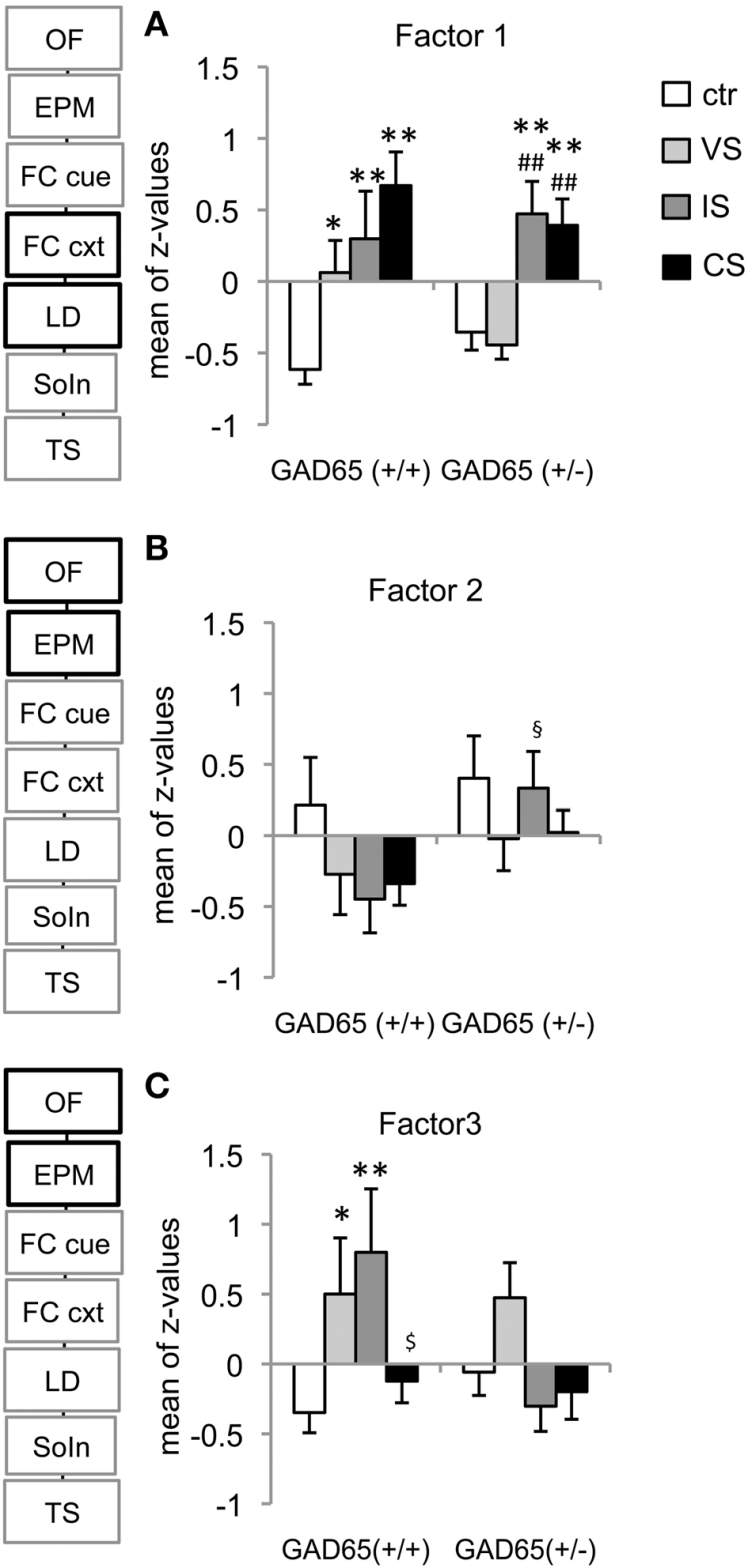
**Identified behavioral domains. (A)** The strongest factor extracted “generalized fear and anxiety,” containing contextual fear memory, post-conditioning anxiety and activity was differentially affected by the genotype and stress experience. In GAD65(+/+) mice any of the stress-protocols induced PTSD-related symptoms. Heterozygous VS-mice fully recovered from juvenile stress experience and show increased behavioral responsiveness only after IS or CS. **(B)** The second extracted factor “preconditioning activity” combined distance (OF) and total arm entries (EPM) and revealed a social isolation-induced reduction in activity in wild type compared to heterozygous mice. **(C)** The third extracted factor, “preconditioning anxiety” combined center time (OF) and % open arm entries (EPM) and confirmed the different responsiveness of GAD65(+/+) mice to each of the stressors alone and a combination of both. ^*^p < 0.05, ^**^p < 0.01 compared to ctr of the same genotype, ^##^*p* < 0.05 compared to VS of the same genotype, ^§^*p* < 0.05 compared to GAD65(+/+) of the same stress group, ^$^*p* < 0.05 compared to IS of the same genotype.

The first extracted factor accounts for 17.91% of total variance and is composed of contextual fear memory (freezing), anxiety (% activity in the light) and total activity in the LD-test (Table 1). Z-transformed “activity in the light” and “total activity” −values were multiplied by (−1) before being combined with contextual freezing, such that increased z would reflect increased fear/anxiety. A significant group effect [*F*_(3, 80)_ = 13.998, *p* < 0.001], but no effect of genotype [*F*_(1, 80)_ = 0.44, *p* = 0.509] or genotype x stressor interaction effects [*F*_(3, 80)_ = 2.08, *p* = 0.109] were obtained; thus *post-hoc* LSD-tests were carried out individually for each genotype. For GAD65(+/+) mice, all three stress groups showed significantly increased scores compared to controls (VS: *p* = 0.028, SI: *p* = 0.006, CS: *p* < 0.001). For GAD65(+/−) mice, however, the VS-group was indistinguishable from controls (*p* = 0.653), while the other two stress groups showed increased scores compared to both control and VS groups (IS vs. control: *p* = 0.001, CS vs. control: *p* = 0.001, IS vs. VS: *p* < 0.001, CS vs. VS: *p* < 0.001) (Figure 4A). This analysis confirms the gradually increased response depending on the stress intensity in wild type mice and a bisectioning of the response in heterozygous mice depending on social interaction with littermates.

#### Factor 2: pre-conditioning activity

The second extracted factor comprised measures for activity collected in the open field and elevated plus maze before the fear conditioning in adulthood. A significant effect was observed only for the genotype [TWA, *F*_(1, 81)_ = 4.303, *p* = 0.041], whereas the stressors led to a uniform, but not significant reduction in activity in GAD65(+/+) mice. Only social isolation induced a significant effect [*t*_(15)_ = −2.227, *p* = 0.042], reducing activity in wild type mice, but not in heterozygotes (Figure 4B).

#### Factor 3: pre-conditioning anxiety

The third extracted factor comprised anxiety-related behaviors collected in the open field (center time) and elevated plus maze (open arm entries) before the fear conditioning in adulthood. The overall effects in this factor are in line with the observations in the elevated plus maze, with a significant effect of stress group [*F*_(3, 79)_ = 3.592, *p* = 0.014]. *Post-hoc* comparisons revealed a significant difference from control mice for the VS− (*p* = 0.04) and the IS group (*p* = 0.01) of wild type mice. Moreover, GAD65(+/+) IS mice differed from CS mice (*p* = 0.043) (Figure 4C).

#### Factor 4: depression-like behavior

The fourth extracted factor comprised measures of social withdrawal and depression in the social interaction and tail suspension tasks. A significant group effect was observed [TWA, *F*_(3, 80)_ = 5.586, *p* = 0.002]. In GAD65(+/+) mice the IS group with an increased depression score differed significantly from controls (*p* = 0.026) and VS−mice (*p* = 0.013). The CS mice showed less of an increase, but still differed from VS (*p* = 0.036). GAD65(+/−) mice showed similar trends, but failed to reach significance.

#### Factor 5: cued fear memory

The fifth extracted factor includes both cue-specific and generalized auditory fear memory. The combined factor revealed no statistically significant impact of genotype or stress exposure [genotype: *F*_(1, 77)_ = 2.938, *p* = 0.091; stressor type: *F*_(3, 77)_ = 0.607, *p* = 0.613; genotype × stressor type: *F*_(3, 77)_ = 1.083, *p* = 0.361].

## Discussion

In the current study we utilized GAD65 haplodeficient mice to investigate the importance of the postnatal maturation of the GABAergic system for the development of traumatic stress induced pathology. The biological, behavioral, cognitive and emotional outcomes of severe stress, particularly when triggered early in development, are multifaceted and complex. So is the clinical picture of psychopathologies like PTSD, with symptoms ranging from memory disturbances, anxiety, mood disturbance and social numbing and with one or the other symptom being more or less pronounced in each particular individual. To comprehensively determine the mutant's response to traumatic stress we (1) applied and combined two different established models of risk factors for stress-induced psychopathologies that have been shown to affect GABAergic function and (2) examined the behavioral outcome with a set of behavioral tests covering various relevant behavioral domains. The five factors statistically extracted from this analysis together account for more than 70% of the total behavioral variability and are in line with validated behavioral measures. For instance, open arm entries in the elevated plus maze and center exploration in the open field loaded onto the same factor, called “pre-conditioning anxiety.” However, in the course of the study it also became evident that sensitivity to the stress procedure varied greatly with respect to the behavioral parameters analyzed and with respect to the GAD65 genotype. We therefore considered both, the single behavioral measures and their combined score in each extracted factor for the analysis of stressor- and genotype effects, which confirmed the existence of profound differences in the development of PTSD-related symptoms between the genotypes.

The common behavioral change across models and strongest behavioral component extracted from our data (factor 1), involves a change in contextually generalized fear and fear conditioning-induced anxiety. Increased anxiety and an overrepresentation of contextual memory components in fear conditioning experiments are frequently reported in PTSD (Pibiri et al., 2008; Bazak et al., 2009; Herrmann et al., 2012; Albrecht et al., 2013) and are particularly pronounced when a previous trauma presentation took place in juvenility (Cohen et al., 2007). Moreover, a tight correlation between stress-induced unconditioned anxiety and conditioned fear has been established (Lukkes et al., 2009). Of note, this factor in our experiments contained only anxiety and activity measures recorded after the adulthood fear conditioning challenge, suggesting that a combination of sensitization stress and adult trauma is required to mimic this behavioral manifestation of PTSD (Bazak et al., 2009). Also, we observed a gradual increase in this factor depending on stress severity, thus fulfilling an important quality criterion of rodent PTSD models (Siegmund and Wotjak, 2006).

In line with previous studies (Stork et al., 2003; Bergado-Acosta et al., 2008), genotype differences between unstressed control mice were never observed. However, within-genotype comparison revealed that GAD65 haplodeficiency provides resilience to juvenile stress-induced changes in generalized fear and conditioned fear induced anxiety, as these animals unlike wild types were undistinguishable from unstressed animals. This is in sharp contrast to the increased susceptibility for PTSD-like behavior of homozygous GAD65 mutants (Bergado-Acosta et al., 2008; Sangha et al., 2009). However, the contextually generalized fear in our current experiments clearly segregated from cued fear memory and was not associated with increased flight or the auditory generalization previously found in GAD65(−/−) mice (Stork et al., 2003; Bergado-Acosta et al., 2008). Thus, the protective effect of GAD65 haplodeficiency appears to be fundamentally different from the phenotype of homozygous mutants. It is likely that the delayed maturation of the GABAergic system in GAD65(+/−) mice (Stork et al., 2000a) may interfere with adaptive changes of the GABAergic system that are induced by the variable juvenile stress (Jacobson-Pick et al., 2008, 2012).

However, this protective effect was not evident in the paradigms involving social isolation. As one explanation, this discrepancy may relate to the different time windows and thus intensity of stress exposure in the applied paradigms, as the social isolation extends beyond the phase of GABAergic deficiency in GAD65(+/−) mice. From a different perspective, it may also be argued that social interactions are required for GAD65 haplodeficiency to exert its protective effect. We have previously shown that in contrast to homozygous mutants unstressed GAD65(+/−) mice resemble their wild type littermates in a set of anxiety and depression tests, hence ruling out *a priori* differences in emotionality as a cause for the observed difference. However, the mutants display significantly fewer attacks and slightly increased grooming behavior in the male intruder aggression test (Stork et al., 2003), indicating a change of social behaviors that may also translate into a different response to social isolation.

In fact, a protective effect of GAD65 haplodeficiency could also be observed upon social isolation. In socially isolated wild type mice we observed a reduced exploration behavior in an open field compared to unstressed mice, resembling previous observations in rats (Avital et al., 2006). However, studies in mice rather report hyperactivity (Pietropaolo et al., 2008a,b) or no changes compared to group-housed animals (Varty et al., 2006; Naert et al., 2011). Moreover, an enhanced exploration of open arms occurred in the elevated plus maze in our experiments, which may reflect an increased arousal response as previously been observed in chronically stressed C57BL/6 mice (Mozhui et al., 2010) and stress-sensitive mutant mice (Stork et al., 2000b). Thus, social isolation stress in wild type mice altered activity and anxiety-levels preceding the adult fear conditioning. GAD65 haplodeficiency prevented both changes effectively, but did not affect these behavioral parameters in juvenile stressed animals. Interestingly, the combination of both stressors alleviated the hyperarousal response of wild types, indicating a process of adaptive resilience, similar to beneficial effects of brief maternal separation (McIntosh et al., 1999; Ricon et al., 2012).

Thus, animals in our study revealed sensitivity to associative (i.e., fear conditioning-related) and non-associative behavioral disturbances (such as anxiety and activity), fulfilling another criterion for PTSD-models as claimed by Siegmund and Wotjak (Siegmund and Wotjak, 2006). The responsiveness to different PTSD-inducing protocols and the selective resilience conferred by the GAD65 haplodeficiency are in line with an independence of contextual generalization and unconditioned hyperarousal reported in another model of PTSD (Sauerhöfer et al., 2012).

Both fear generalization and arousal further segregated from depression-like and social withdrawal symptoms (factor 4). Depression symptoms and social numbing are frequently observed in PTSD patients and the individual difference in fear and depression symptom profile has led to the definition of posttraumatic depression as a particular form of trauma-induced disease. Indeed, wild type animals in the social isolation group but not those in the juvenile stress group showed an increase in the combined score, indicating a moderate depression-like behavioral change. This is in line with a previous report of Tsoory et al. (2007), who reported brief, intensive stress similar to ours to be preferentially associated with an anxiety cluster and not with a depression cluster (Tsoory et al., 2007). In contrast, social isolation induces both depression and anxiety, but via different mechanisms (Wallace et al., 2009). Hippocampal GABA- and GAD65 levels are reduced in mouse models of depression shortly after the end of chronic mild stress (Garcia-Garcia et al., 2009), as well as weeks thereafter (Elizalde et al., 2010). However, GAD65 mutation in our experiments had no effect on this extracted factor, as also observed previously in naïve mutants (Stork et al., 2000a). This suggests a selective involvement of GAD65 in fear- and arousal, but not depression-related mechanisms in the stress models employed here.

The involvement of GABA in the stress response and stress-related psychiatric disorders is well established (for review see Kalueff and Nutt, 2007), but its precise role is still controversial. In patients, most studies report a negative correlation between the GABAergic tone and symptom severity (Vaiva et al., 2004, 2006), but Girard et al. (2007) identified benzodiazepine application as a risk factor for PTSD development in in-patients of an intensive care unit (Girard et al., 2007). In rodents, social isolation (Pibiri et al., 2008), variable stress (Poulter et al., 2010) as well as fear conditioning (Heldt and Ressler, 2007; Rea et al., 2009) have been shown to induce changes in different GABAergic factors. We have previously assembled evidence that null mutation of GAD65 results in a PTSD-like behavioral phenotype in fear conditioned mice (Stork et al., 2003; Bergado-Acosta et al., 2008; Sangha et al., 2009) and Heldt and Ressler (2007) showed a correlation of intra-amygdalar GAD67 expression with the level of conditioned fear in rats (Heldt and Ressler, 2007). In contrast, Tasan et al. (2011) found increased GAD65- and GAD67 levels in the amygdala of a high anxiety mouse strain (Tasan et al., 2011). Our current data support the ambivalent role of GAD65 and reconcile previous findings by demonstrating a protective effect of GAD65 haplodeficiency toward PTSD-related symptoms induced by juvenile stress and/or social isolation.

## Author contributions

Iris Müller, Kunihiko Obata, Gal Richter-Levin, and Oliver Stork designed the study, and discussed results. Iris Müller performed the experiments and analyzed the data. Iris Müller and Oliver Stork wrote the paper. Kunihiko Obata and Gal Richter-Levin critically reviewed the manuscript and gave valuable intellectual input.

### Conflict of interest statement

We did not receive payments or services from a third party for any aspect of the submitted work. We do not have financial relationships with entities influencing our work. There are no patents, copyrights or royalties relevant to this paper. Further, there are no activities or relationships that influenced or could give the appearance of potentially influencing the present work. The authors declare that the research was conducted in the absence of any commercial or financial relationships that could be construed as a potential conflict of interest.
