# Supplementary material for: GAD65 haplodeficiency conveys resilience in animal models of stress-induced psychopathology
Source: Front Behav Neurosci. 2014 Aug 7;8:265. doi: 10.3389/fnbeh.2014.00265 (PMC4124590; doi:10.3389/fnbeh.2014.00265)
Supplement: Supplementary file 1 [file DataSheet1.PDF]

Supplementary Material

GAD65 haplodeficiency conveys resilience in animal models of stress-induced psychopathology

Iris Müller[1], Kunihiko Obata[2], Gal Richter-Levin[3] and Oliver Stork[1,4,*]

[1] Department of Genetics & Molecular Neurobiology, Institute of Biology, Otto-von-Guericke University, Magdeburg, Germany
[2] National Institute for Physiological Sciences, Okazaki, Aichi, Japan
[3] Department of Neurobiology and Ethology and Department of Psychology, University of Haifa, and Institute for the Study of Affective Neuroscience Haifa, Israel
[4] Center for Behavioural Brain Sciences, Magdeburg, Germany

***Correspondence:** Prof. Dr. Oliver Stork, Department of Genetics & Molecular Neurobiology, Institute of Biology Otto-von-Guericke-University, Magdeburg, Leipziger Str. 44 (Haus 91), 39120 Magdeburg, Germany
oliver.stork@ovgu.de

1. Supplementary Data

Supplementary Table 1: Factor analysis with Varimax rotation. Five independent factors were extracted that together accounted for 70.079 % variability of the data. Extraction Method: Principal Component Analysis. V. = variance, cum. = cumulative

Total Variance Explained

Component	Initial Eigenvalues			Extraction Sums of Squared Loadings			Rotation Sums of Squared Loadings		
	Total	% of V.	Cum. %	Total	% of V.	Cum. %	Total	% of V.	Cum. %
1	2.544	23.124	23.124	2.544	23.124	23.124	1.958	17.803	17.803
2	1.539	13.994	37.119	1.539	13.994	37.119	1.706	15.514	33.317
3	1.277	11.611	48.729	1.277	11.611	48.729	1.428	12.979	46.295
4	1.246	11.331	60.06	1.246	11.331	60.06	1.323	12.027	58.323
5	1.102	10.019	70.079	1.102	10.019	70.079	1.293	11.757	70.079
6	0.788	7.16	77.239						
7	0.736	6.693	83.931						
8	0.638	5.798	89.729						
9	0.513	4.662	94.391						
10	0.357	3.243	97.634						
11	0.26	2.366	100						

Supplementary Table 2: Factor loadings after Quartimax rotation. Quartimax validated the factors extracted by Varimax, since the variables segregated into the same factors. Factor loadings above 0.5 are highlighted in bold. Extraction Method: Principal Component Analysis. Rotation Method: Quartimax with Kaiser Normalization, rotation converged in 7 iterations.

QUARTIMAX-rotation

	Rotated Component Matrix				
	1	2	3	4	5
OF: distance (m)	0.113	**0.866**	-0.11	-0.071	-0.05
OF: center time (s)	-0.155	0.012	**0.801**	-0.093	0.003
EPM: total arm entries	0.071	**0.833**	0.028	-0.056	0.085
EPM: % open arm entries	0.365	-0.066	**0.671**	0.211	-0.109
FC: shock context_freezing (s)	**-0.53**	-0.235	0.409	0.251	0.059
FC: CS- freezing (s)	0.015	0.087	-0.127	0.086	**0.873**
FC: CS+ freezing (s)	-0.442	-0.223	0.307	-0.082	**0.508**
LD: % activity in light	**0.865**	-0.137	-0.012	-0.186	0.114
LD: total activity (s)	**0.734**	0.315	0.191	0.115	-0.096
SI: % time of mouse contacts	-0.011	0.057	-0.046	**-0.818**	-0.282
TS: time immobile (s)	-0.244	-0.11	0.026	**0.672**	-0.373
